# A Brief Intervention for Malnutrition among Older Adults: *Stepping Up Your Nutrition*

**DOI:** 10.3390/ijerph17103590

**Published:** 2020-05-20

**Authors:** Matthew Lee Smith, Caroline D. Bergeron, Sue Lachenmayr, Leigh Ann Eagle, Judy R. Simon

**Affiliations:** 1 Center for Population Health and Aging, Texas A&M University, College Station, TX 77843, USA; caroline.bergeron@inspq.qc.ca; 2 School of Public Health, Texas A&M University, College Station, TX 77843, USA; 3 College of Public Health, The University of Georgia, Athens, GA 30602, USA; 4 Institut National de Santé Publique du Québec, Quebec City, QC G1V 5B3, Canada; 5 Maryland Living Well Center of Excellence, Salisbury, MD 21804, USA; bslach@earthlink.net (S.L.); lae2@macinc.org (L.A.E.); 6 Maryland Department of Aging, Baltimore, MD 21201, USA; judy.r.simon@gmail.com

**Keywords:** malnutrition, nutrition risk, falls, fall prevention, intervention, older adults

## Abstract

Despite a multitude of nutritional risk factors among older adults, there is a lack of community-based programs and activities that screen for malnutrition and address modifiable risk among this vulnerable population. Given the known association of protein and fluid consumption with fall-related risk among older adults and the high prevalence of falls among Americans age 65 years and older each year, a brief intervention was created. Stepping Up Your Nutrition (SUYN) is a 2.5 h workshop developed through a public/private partnership to motivate older adults to reduce their malnutrition risk. The purposes of this naturalistic workshop dissemination were to: (1) describe the SUYN brief intervention; (2) identify participant characteristics associated with malnutrition risk; and (3) identify participant characteristics associated with subsequent participation in Stepping On (SO), an evidence-based fall prevention program. Data were analyzed from 429 SUYN participants, of which 38% (*n* = 163) subsequently attended SO. As measured by the SCREEN II^®^, high and moderate malnutrition risk scores were reported among approximately 71% and 20% of SUYN participants, respectively. Of the SUYN participants with high malnutrition risk, a significantly larger proportion attended a subsequent SO workshop (79.1%) compared to SUYN participants who did not proceed to SO (65.8%) (χ^2^ = 8.73, *p* = 0.013). Findings suggest SUYN may help to identify malnutrition risk among community-dwelling older adults and link them to needed services like evidence-based programs. Efforts are needed to expand the delivery infrastructure of SUYN to reach more at-risk older adults.

## 1. Introduction

Nearly 50% older adults are at risk for malnutrition [1], yet routine screening for malnutrition in the community is rare [2]. Malnutrition refers to imbalances in a person’s nutritional intake and/or their body’s capacity to utilize nutrients, which in turn reduces their ability to maintain or repair tissue [3,4]. A person suffering from malnutrition can be identified with two of the following characteristics: insufficient caloric intake, significant weight loss, fluid retention, reduced grip strength, and fat loss [4]. Malnutrition, including vitamin D deficiency and lack of protein, dehydration, and lack of physical exercise, is associated with an increased risk of falls and other serious health conditions such as frailty among older adults [5,6,7,8,9,10,11].

Malnutrition and dehydration are associated with declines in health status [12] and decreased bone health [13] resulting in increased frailty and greater fall risk [14,15], higher rates of hospitalization [16], and increased mortality risk [17,18,19,20]. Considering that between 25% and 33% of older adults age 65 years and older fall every year [21,22], the relationship between malnutrition, dehydration, and falls among older people is important to consider [15,20]. It is well documented that protein intake can prevent the loss of lean muscle mass as people age [23,24]. Therefore, increasing protein intake may be a modifiable behavior to prevent muscle loss, loss of balance, and falls [15,23]. Randomized-controlled trials recommend combining protein intake with physical activity such as resistance training to increase muscle mass and strength [25]. Moreover, hydration helps to regulate blood pressure and helps to prevent weakness and dizziness [21,26], which is also a modifiable behavior to prevent falls [22]. 

Many physical, psychological, social, and economic factors contribute to older adults’ increased risk of malnutrition and dehydration [23]. Loss of muscle mass, decreased kidney function, increased polypharmacy, and decreased thirst sensation as a person ages contribute to having less body water and increase a person’s dehydration [24]. Muscle atrophy also leads to muscle weakness, which can also limit a person’s mobility to buy and prepare food [27]. Having dental problems including periodontal disease, bleeding gums, or a dry mouth can reduce an older person’s ability to swallow or eat certain foods, which restricts their food choices and consumption [28,29]. Quandt and colleagues found that older adults with oral health issues most commonly avoid eating whole fruits, raw vegetables, and meats [29]. Not eating these foods also limits intake of their high-water content. Older adults experience a decreased sense of appetite and thirst [30,31], as well as changes in taste, smell, and vision, which can make eating less enjoyable [30,32]. Having one or more chronic conditions and taking medications to manage these diseases can also reduce appetite [30,33]. 

Among the various psychological and social factors, memory impairment may result in older adults forgetting or refusing to eat or stay hydrated because of dysphagia [34,35,36]. Older adults who suffer from depression, who are lonely, or socially isolated may have greater risks of eating alone, not staying hydrated, or not being able to prepare healthy foods [37]. Economic and transportation issues can also reduce older people’s food accessibility and their ability to maintain a nutrient-dense diet [37,38,39,40].

To date, there are no health promotion and disease prevention programs that specifically address older adult malnutrition and meet the Administration for Community Living’s criteria for an evidence-based program [41]. While some of these programs incorporate nutrition or dietary selection issues, these are often in the context of disease self-management (e.g., specifically for diabetes) or general wellness [42]. Moreover, few evidence-based nutritional interventions specifically aim to reduce falls and fall-related risk [43], which is true even among the few that target malnutrition and dehydration among older adults [44]. 

Therefore, until a specific intervention is developed, evaluated, and documented to address malnutrition among older adults, brief interventions may have great potential to raise awareness about malnutrition, modify risk behaviors, and offset possible falls among older adult populations. Such brief interventions can be offered independently or as introductory workshops—also termed a “Session Zero”—for an evidence-based program (e.g., fall prevention, disease self-management) [45]. As documented in the aging services network, offering a Session Zero to complement evidence-based workshops can be helpful to orient participants to the topic and process, alleviate time restrictions associated with data collection, provide additional content not covered in the evidence-based program, and assist in the attendance and retention rates of program participants [45].

Taking this into account, the purposes of this study were to: (1) describe a brief intervention for malnutrition, Stepping Up Your Nutrition (SUYN); (2) identify participant characteristics associated with malnutrition risk; and (3) identify participant characteristics associated with subsequent participation in Stepping On (SO), an evidence-based fall prevention program.

## 2. Materials and Methods 

### 2.1. Workshops

Stepping Up Your Nutrition (SUYN) is an interactive group-based workshop developed to help older adults remain independent, increase awareness about the link between malnutrition and falls risk, and prevent falls-related admissions. Developed by a team of registered dietitians and experts on health and aging, the key messages introduced in SUYN include: (1) how nutrition and muscle strength impact falls risk; (2) exercise, fluids, and protein maintain and build strong muscles; and (3) nutrition-focused actions to reduce falls risk. SUYN utilizes strategies to de-stigmatize malnutrition, underscore muscle strength loss with age, and provide solutions to lessen muscle loss by increasing fluids and protein. Workshops are led by certified lay leaders who have undergone a formal 2.5 h training (face-to-face or online) and utilize a standardized leader manual. The leader training is ideal for peers training in evidence-based programs (EBP), community health workers, and community-based organization staff. During the 2.5 h workshop, participants engage in role play and problem-solving activities, complete planning tools to increase liquids and proteins, learn to read food labels, and make an action plan. At the beginning of the workshop, participants complete a nutrition assessment as well as a baseline questionnaire. Mid-way through the workshop, a break is given where participants are introduced to (and taste) protein-rich foods and drinks (e.g., nutritional supplements). Collectively, SUYN content and activities were designed to give participants the skills and confidence to make changes regarding their nutrition and fall-related risk. Because nutrition is strongly linked to fall prevention and chronic disease self-management, SUYN was initially designed as a Session Zero to be held in conjunction with other evidence-based programs that focus on these topics specifically. However, based on the unique content of SUYN, the brief intervention is suitable for stand-alone delivery in group or one-on-one formats. An outline of the SUYN brief intervention is provided in Table 1.

In this naturalistic workshop dissemination, SUYN participants may have elected to attend Stepping On (SO), an evidence-based fall prevention program [46,47]. SO is a group-based intervention originally developed in Australia [48] and adapted by the Wisconsin Institute for Healthy Aging for use in the United States [49,50]. The program includes discussions about vitamin D, bone strength, medications that increase falls risk, as well as the importance of physical activity. During workshop sessions, participants engage in a variety of appropriate balance and weight-bearing exercises. Through a randomized-controlled trial, SO has been shown to reduce falls among older adults at risk for falling [48]. The 7-week intervention is led by trained leaders; health professionals are invited as guest experts [49]. SO workshop sessions are two hours in duration and held once a week for seven consecutive weeks.

### 2.2. Participants and Procedures

*SUYN Leader Training*. SUYN is facilitated by one or two trained lay leaders or healthcare professionals who are currently credentialed to lead an evidence-based program (e.g., fall prevention, disease self-management). Training sessions include detailed processes for data collection in terms of consenting participants, collecting nutrition risk assessments, and performing handgrip strength (if the site has the equipment and resources to do so). Using the SUYN curriculum, facilitators are educated about the brief intervention content and given opportunities to practice their facilitation and role-playing skills. Further, facilitators receive training about how to interact with older adults and linking them to available and needed nutritional services and resources. Quality assurance, program fidelity, and adherence to the program curriculum and protocols were monitored through leader observation during at least one onsite visit by a SUYN Master Trainer. A total of 29 possible trained facilitators were asked to deliver at least two SUYN workshops for 10–16 participants annually.

*Recruitment*. For this naturalistic community-based dissemination, SUYN workshops were delivered in a variety of community and clinical locations across the state of Maryland. Overall, 48 SUYN workshops were delivered across eight cities in 10 distinct ZIP Codes. Based on the trained leader infrastructure described above, SUYN was delivered in 42 unique sites aligned with the national dissemination of evidence-based programs [51,52], which included Area Agencies on Aging, senior centers, healthcare organizations, recreation facilities, residential facilities, low-income housing facilities, and faith-based organizations. For SO, workshops were offered in a total of 22 unique sites in 13 cities (in 20 distinct ZIP Codes). A total of six organizations offered SUYN+SO. However, participants were not required to attend SUYN and SO at the same location.

A total of 429 SUYN participants were included in this naturalistic workshop dissemination, of which 38.0% (n = 163) subsequently attended SO. Because different data collection instruments are used for SUYN and SO, and only a portion of participants attended both workshops, a series of analyses were performed using the SUYN data collection instrument only. Given missing data differed across variables, comparisons were made pairwise to assess differences (counts for each set of analyses are reported to describe the proportion of participants included in each comparison). 

### 2.3. Measures

*SUYN: Baseline Malnutrition Risk Screening*. Although a variety of nutrition risk screenings exist for older adults [53,54], this SUYN demonstration used the SCREEN II^®^ because of its applicability to the population and distinct scoring mechanism [55,56,57]. This instrument is considered a valid malnutrition screening tool for community settings [53,57], given nutritional assessments administered to older adults can rapidly identify their malnutrition risk [53]. The SCREEN II^®^ consists of 14 multi-part items that assess an older adult’s perceptions of weight and appetite, diet composition, and barriers to eating and cooking. Using the predetermined scoring mechanism, scores of 55 and higher indicate no or low nutrition risk, scores of 50 to 54 indicate moderate risk for malnutrition, and scores of 49 and lower indicate high malnutrition risk [55]. 

*SUYN: Baseline Characteristics*. Participants enrolled in SUYN were asked to report information about themselves prior to attending the brief intervention. Participants self-reported their age and gender. They reported whether they had fallen in the past three months and their fear of falling. Participants also reported their dietary behaviors and perceptions by responding to items regarding their weight change over the past month, self-described appetite, eating with others, difficulties getting groceries, skipping meals, and knowing about resources to overcome financial challenges for getting food. (See Table 2 and Table 4).

*SUYN: Baseline Knowledge and Confidence*. Participants enrolled in SUYN were asked to report their knowledge and confidence specifically related to content presented in the brief intervention. Five knowledge-based items asked participants to self-report if they knew how much protein and fluid should be consumed daily, if they understood their nutrition risk and ways to improve it, and if they understood the importance of nutrition and muscular strength to prevent falls. Each of these items were asked on Likert-type scales ranging from 1 to 5, with higher responses indicating higher self-proclaimed knowledge. Six confidence-related items asked participants to self-report their ability to identify foods that are good sources of protein, identify recommended portion sizes, and identify ways to get healthy foods. They were also asked to report if they could list ways to increase fluid intake, read food labels, and set healthy eating goals. Each of these items were asked on Likert-type scales ranging from 1 to 4, with higher responses indicating higher self-proclaimed confidence. All knowledge- and confidence-related items were analyzed continuously to improve interpretations based on the number of response categories (See Table 3).

### 2.4. Statistical Analyses

All analyses were performed using SPSS version 25. Based on the naturalistic enrollment of participants in these interventions, common data elements were not collected uniformly from all study participants. A series of descriptive and bivariate analyses were executed based on the common data elements between the intervention condition (i.e., SUYN Only vs. SUYN + SO). Descriptive statistics were performed, which were then compared by intervention conditions and nutrition risk scores at baseline. When describing SUYN Only vs. SUYN + SO, categorical variables were compared using chi-square tests and continuous variables were compared using independent sample *t*-tests and one-way ANOVA. Missing values were excluded case wise, and statistics were reported for non-missing data only.

## 3. Results

### 3.1. Baseline SUYN Characteristics by Malnutrition Risk and Dietary Behavior Factors

A total of 62% of participants enrolled in SUYN Only, while another 38% enrolled in SUYN + SO. Among SUYN participants who also attended SO, the average time between interventions was 19.48 (±64.36) days [range from 0 days to 393 days]. The average age of SUYN participants was 74.71 (±11.45) years, with 33.7% being age 80 years or older. The majority of participants (63.3%) was female. Approximately 71% of SUYN participants scored at high malnutrition risk on the SCREEN II, followed by 19.6% scoring at moderate risk, and 9.6% scoring at no/low risk. About one-in-five (21.1%) SUYN participants reported a fall within the past three months, and 27.2% and 16.5% of participants reported being “somewhat” and “a lot” fearful of falling, respectively. Over half of participants (52.2%) reported never/rarely/sometimes eating meals with others daily, while 41.3% self-described their appetite as poor/fair/good, and 49.6% sometimes/often skipped meals. Twenty percent of participants reported sometimes/often/almost always having problems getting groceries, and 16.2% reported their groceries did not last and they did not have money to purchase more. 

When comparing SUYN participant characteristics by malnutrition risk score, a significantly larger proportion of SUYN participants who also participated in SO scored at high malnutrition risk on the SCREEN II (χ^2^ = 8.73, *p* = 0.013). A significantly larger proportion of females scored at high malnutrition risk (χ^2^ = 10.26, *p* = 0.006). Larger proportions of those reporting high malnutrition risk also reported eating meals with others less regularly (χ^2^ = 23.86, *p* = 0.001), a poorer appetite (χ^2^ = 18.48, *p* = 0.001), skipping meals more often (χ^2^ = 36.40, *p* < 0.001), and not having money to purchase more groceries when groceries did not last (χ^2^ = 13.50, *p* = 0.009). (See Table 2).

Generally, SUYN participants self-reported moderate to high knowledge and confidence about nutrition and fall prevention at baseline. Scores for the five knowledge items ranged from 3.33 (±1.14) to 3.94 (±1.11). On average, highest knowledge was reported for “understand the importance of muscle strength to prevent falls,” while lowest knowledge was reported for “know how much protein I should consume daily to meet my needs.” Scores for the seven confidence items ranged from 3.05 (±0.76) to 3.93 (±1.06). On average, highest confidence was reported for “can identify foods that are good sources of protein,” while lowest confidence was reported for “can identify recommended portion sizes for different foods.”

When comparing SUYN participant baseline knowledge and confidence by malnutrition risk score, participants with higher malnutrition risk scores consistently reported lower knowledge and confidence. On average, participants with higher malnutrition risk reported significantly lower knowledge on four of the five items (*p* < 0.05). Similarly, on average, participants with higher malnutrition risk reported significantly lower confidence on five of the seven items (*p* < 0.05). Two of the four non-significantly different items (one for knowledge, one for confidence) surrounded fluid intake (i.e., “know how much fluid I should consume daily to meet my needs” and “can list ways to increase fluid intake”) (See Table 3). 

### 3.2. Baseline Malnutrition Risk for SUYN Participants by Study Condition

Table 4 compares baseline malnutrition risk for SUYN participants by whether they only attended SUYN or attended SUYN + SO. On average, SUYN + SO participants were over five years older than SUYN Only participants (t = −5.39, *p* < 0.001). A significantly larger proportion of SUYN + SO participants scored at high malnutrition risk compared to SUYN Only participants. A significant difference was also observed for fear of falling between the groups (i.e., a smaller proportion of SUYN + SO participants reported “not at all” and a larger proportion reported “somewhat” compared to SUYN Only participants) (See Table 4).

## 4. Discussion

This study described the SUYN brief intervention for identifying malnutrition risk among community-based older adults. The screening element at the beginning of the intervention showed promise as a necessary first step to identify older adults with malnutrition risk, who may otherwise not be screened in the community. In the US, malnutrition screenings are typically provided to older adults who present at the clinical setting, but they are rarely performed in community settings [58]. While SUYN may reach some of this vulnerable population, it also serves older adults not necessarily reached by these settings and services. Therefore, SUYN can help identify malnutrition risk that may otherwise go undetected within the evidence-based program movement in the US.

Conversely, because SUYN is delivered to older adults in community-based settings, recruitment is not limited to individuals with documented malnutrition or fall-related risk or fear. To assess the potential impact of SUYN on health outcomes, including falls in future studies, more purposive targeting is warranted to recruit and engage more at-risk participants with fear of falling or malnutrition risk.

As a stand-alone brief intervention, SUYN shows promise in the evidence-based program movement by linking participants who had lower knowledge about nutrition and fall prevention, higher malnutrition risk, and greater fear of falling to subsequent SO fall prevention workshops. As a Session Zero, SUYN also provided additional information about the purpose, content, and expectations of SO [45], which may have highlighted the value and importance for these at-risk participants to engage in subsequent SO workshops. 

Results revealed that participants with poor nutrition knowledge had higher malnutrition risk. This finding has been reported in previous studies. For example, Kikafunda and Lukwago [59] suggested that low nutritional knowledge played a role in the poor nutritional health status of older participants. In their qualitative study, Koo and colleagues [60] also reported that lack of nutritional knowledge and simply not knowing one’s poor nutritional status may have contributed to older adult malnutrition. Alternatively, in Turconi et al.’s study [61], even though the majority of their independent older adults had good nutritional knowledge, only about 30% adopted a healthy diet. While increasing education may not directly lead to healthy behaviors, this finding reiterates the importance of education as a modifiable risk factor for malnutrition. 

Senior nutrition programs implemented by the Older Americans Act are required to provide nutrition education [61]; SUYN could be offered as a single class, or its content can be broken down and delivered across several shorter sessions. It can be delivered in groups or in one-on-one settings with more vulnerable populations (e.g., in-home, residential facilities). Evidence-based program leaders, community health workers, healthcare professionals, and volunteers can be trained to deliver SUYN and administer malnutrition screening to their older adult clients, which has the potential to greatly expand malnutrition screening in community settings. While this community-based dissemination demonstration only utilized the in-person SUYN training, the training has since been translated for online delivery to expand the training’s access and reach nationwide. Despite the preferred training format, receiving SUYN training can enable those who work with older adults to use the information in versatile ways and contexts within their regular workflow. Such contexts can vary broadly from a group presentation in a faith-based organization to a one-on-one intervention to a homebound older adult (e.g., formally or informally deliver SUYN content by lay health workers such as Meals on Wheels volunteers or community health workers) [62,63].

The SUYN brief intervention was designed to address malnutrition and fall-related risk and was examined in the context of subsequently enrolling in and attending an evidence-based fall prevention program (i.e., SO) for this study. However, SUYN’s content could also apply to other evidence-based programs related to physical activity and chronic disease self-management education (CDSME). Currently, many CDSME programs that emphasize the importance of nutrition neither screen for nor address malnutrition and dehydration [38]. Future efforts should consider linking SUYN to a variety of evidence-based programs for falls, disease self-management, physical activity, and other topics.

Despite the benefits seen in this study, some limitations are worth noting. First, this pragmatic trial used a naturalistic dissemination of SUYN and SO in one state and may not be generalized to other states. Second, all data were self-reported, which may have introduced reporting or recall bias. Additional potentially important variables and descriptors associated with older adult risk for malnutrition and falls may not have been collected (e.g., number of chronic conditions, race/ethnicity, income), which may hinder interpretation of findings. Further, the malnutrition risk assessment and associated knowledge and confidence screeners used in this study are not generally deployed within the general public, which makes it difficult to generalize or compare aspects of this sample to other older adult populations. Third, while protein and fluid consumption were the focus of the educational elements of SUYN because of their relationships to falls among older adults, this brief intervention did not address all aspects of malnutrition such as vitamins and minerals needed at varying life stages (e.g., calcium for bone health). Future efforts may consider expanding SUYN to become a multi-session stand-alone workshop that more comprehensively addresses malnutrition among older adults. Further, the dissemination of this program was limited to one state because of the in-person delivery infrastructure; therefore, efforts are needed to expand the SUYN training infrastructure and use complementary delivery modes (e.g., online workshops) to directly reach the older adults.

## 5. Conclusions

This study described the SUYN brief intervention, identified participant characteristics associated with malnutrition risk, and identified participant characteristics associated with subsequent participation in SO. Overall, SUYN was a successful community-based brief intervention to identify baseline malnutrition risk, which may have otherwise gone unrecognized and unaddressed. Brief interventions have a role in the evidence-based movement to introduce older adults in informative sessions and link the most at-risk participants to subsequent falls prevention workshops.

## Figures and Tables

**Table 1:** Stepping Up Your Nutrition (SUYN) Brief Intervention Overview.

Components	Description
Introduction	Overview of the workshop and expectations; introduction of facilitators and participants
Baseline Data Collection	Consents (if needed); Pre-Test (knowledge, risk)
Nutrition Affects Falls	Discuss and share beliefs about why food is important
Muscle Matters	Overview of muscles and changes at different life stages; participants share why less muscle increases risk of falls
Nutrients to Know	Identify which food has most protein and how much protein is needed; discuss strategies about how to get more protein throughout the day; label reading activity; discuss the importance of hydration and impact of dehydration; brainstorm how to drink more fluid
Measuring Hand Grip Strength (Optional)	Measure participant grip strength following specific protocol (if resources exist)
Break	Offer food and protein drink tasting (dispel myths and encourage diversifying existing food/drink consumption)
Personalized Nutrition Risk Score	Conduct role-play so participants can share strengths and risks in the example and rate their nutrition habits; score personalized risk score and obtain interpretation of their risk (stoplight); identify community and clinical resources based on risk level
Action Planning	Facilitator identifies the components of an action plan and demonstrates how to create one action plan; participants make action plan and document it; participants share action plan with the group; participants write nutrition risk score, handgrip strength score (if collected), and action plan on Doctor Letter

**Table 2:** Baseline Characteristics for SUYN Participants by Malnutrition Risk Score.

Variables	TOTAL	SCREEN II			χ^2^	*p*-Value
		NO/LOW	MODERATE	HIGH		
Intervention (*n* = 429)					8.73	0.013
SUYN Only	266 (62.0%)	30 (73.2%)	61 (72.6%)	175 (57.6%)		
SUYN + SO	163 (38.0%)	11 (26.8%)	23 (27.4%)	129 (42.4%)		
Age Group (*n* = 383)					10.85	0.210
64 Years and Younger	51 (13.3%)	2 (6.3%)	15 (19.5%)	34 (12.4%)		
65–69 Years	54 (14.1%)	2 (6.3%)	11 (14.3%)	41 (15.0%)		
70–74 Years	66 (17.2%)	10 (31.3%)	11 (14.3%)	45 (16.4%)		
75–79 Years	83 (21.7%)	7 (21.9%)	19 (24.7%)	57 (20.8%)		
80 Years and Older	129 (33.7%)	11 (34.4%)	21 (27.3%)	97 (35.4%)		
Gender (*n* = 229)					10.26	0.006
Male	84 (36.7%)	10 (55.6%)	21 (53.8%)	53 (30.8%)		
Female	145 (63.3%)	8 (44.4%)	18 (46.2%)	119 (69.2%)		
Fallen in Past 3 Months (*n* = 388)					5.76	0.056
No	306 (78.9%)	30 (90.9%)	65 (84.4%)	211 (75.9%)		
Yes	82 (21.1%)	3 (9.1%)	12 (15.6%)	67 (24.1%)		
Fear of Falling (*n* = 429)					5.41	0.492
Not At All	100 (25.7%)	13 (37.1%)	24 (30.0%)	63 (23.0%)		
A Little	119 (30.6%)	11 (31.4%)	25 (31.3%)	83 (30.3%)		
Somewhat	106 (27.2%)	7 (20.1%)	19 (23.7%)	80 (29.2%)		
A Lot	64 (16.5%)	4 (11.4%)	12 (15.0%)	48 (17.5%)		
Weight Changed in Past 30 Days (*n* = 288)					6.35	0.385
Yes, Gained	49 (17.0%)	2 (9.1%)	11 (18.0%)	36 (17.6%)		
No, Stayed Same	189 (65.6%)	19 (86.4%)	42 (68.9%)	128 (62.4%)		
Yes, Lost	43 (14.9%)	1 (4.5%)	7 (11.5%)	35 (17.1%)		
Don’t Know	7 (2.4%)	0 (0.0%)	1 (1.6%)	6 (2.9%)		
Eat Meals with Someone Daily (*n* = 292)					23.86	0.001
Almost Always	90 (30.8%)	14 (63.6%)	27 (43.5%)	49 (23.6%)		
Often	38 (13.0%)	2 (9.1%)	9 (14.5%)	27 (13.0%)		
Sometimes	111 (38.0%)	6 (27.3%)	17 (27.4%)	88 (42.3%)		
Never/Rarely	53 (18.2%)	0 (0.0%)	9 (14.5%)	44 (21.2%)		
Self-Described Appetite (*n* = 290)					18.48	0.001
Very Good	170 (58.6%)	19 (86.4%)	44 (73.3%)	107 (51.4%)		
Fair/Good	108 (37.2%)	3 (13.6%)	16 (26.7%)	89 (42.8%)		
Poor	12 (4.1%)	0 (0.0%)	0 (0.0%)	12 (5.8%)		
Problems Getting Groceries (*n* = 285)					12.59	0.050
Never / Rarely	228 (80.0%)	21 (95.5%)	56 (91.8%)	151 (74.8%)		
Sometimes	42 (14.7%)	1 (4.5%)	4 (6.6%)	37 (18.3%)		
Often	8 (2.8%)	0 (0.0%)	1 (1.6%)	7 (3.5%)		
Almost Always	7 (2.5%)	0 (0.0%)	0 (0.0%)	7 (3.5%)		
Groceries Didn’t Last, Didn’t Have Money for More (*n* = 277)					13.50	0.009
Never	232 (83.8%)	19 (95.0%)	59 (96.7%)	154 (78.6%)		
Sometimes	33 (11.9%)	1 (5.0%)	1 (1.6%)	31 (15.8%)		
Often	12 (4.3%)	0 (0.0%)	1 (1.6%)	11 (5.6%)		
Skipped Meals (*n* = 272)					36.40	<0.001
Never	137 (50.4%)	19 (90.5%)	44 (72.1%)	74 (38.9%)		
Sometimes	111 (40.8%)	2 (9.5%)	16 (26.2%)	93 (48.9%)		
Often	24 (8.8%)	0 (0.0%)	1 (1.6%)	23 (12.1%)		
Know Where to Get Resources, If Not Enough Money for Food (*n* = 249)					1.97	0.741
Often	72 (28.9%)	5 (27.8%)	14 (25.9%)	53 (29.9%)		
Sometimes	48 (19.3%)	3 (16.7%)	8 (14.8%)	37 (20.9%)		
Never	129 (51.8%)	10 (55.6%)	32 (59.3%)	87 (49.2%)		

**Table 3:** Knowledge and Confidence for SUYN Participants by Malnutrition Risk Score.

Variables	TOTAL		SCREEN II						*f*	*p*-Value
			NO/LOW		MODERATE		HIGH			
	*n*	Mean (SD)	*n*	Mean (SD)	*n*	Mean (SD)	*n*	Mean (SD)		
**Knowledge**										
Know How Much Protein I Should Consume Daily to Meet Needs	262	3.33 (±1.14)	20	4.15 (±1.04)	61	3.54 (±1.09)	181	3.17 (±1.12)	8.46	<0.001
Know How Much Fluid I Should Consume Daily to Meet Needs	265	3.66 (±1.17)	21	4.00 (±1.30)	61	3.87 (±1.15)	183	3.56 (±1.15)	2.59	0.077
Understand Nutrition Risk and Ways to Improve It	267	3.89 (±1.03)	21	4.38 (±0.97)	58	4.14 (±1.08)	188	3.76 (±0.99)	5.92	0.003
Understand the Importance of Nutrition to Prevent Falls	264	3.65 (±1.10)	21	4.38 (±0.97)	60	3.83 (±1.11)	183	3.51 (±1.07)	7.33	0.001
Understand the Importance of Muscle Strength to Prevent Falls	262	3.94 (±1.11)	20	4.40 (±1.00)	62	4.18 (±0.98)	180	3.81 (±1.15)	4.43	0.013
**Confidence**										
Can Identify Foods That Are Good Sources of Protein	280	3.93 (±1.06)	21	4.48 (±0.81)	62	4.05 (±1.29)	197	3.83 (±0.98)	4.11	0.017
Can Identify Recommended Portion Sizes for Different Foods	274	3.05 (±0.76)	20	3.25 (±0.97)	60	3.27 (±0.63)	194	2.96 (±0.76)	4.44	0.013
Can Identify Ways to Get Healthy Foods	275	3.16 (±0.73)	20	3.30 (±0.92)	61	3.36 (±0.52)	194	3.08 (±0.75)	4.05	0.019
Can List Ways to Increase Fluid Intake	261	3.70 (±1.09)	20	3.85 (±1.23)	61	3.97 (±0.95)	180	3.59 (±1.10)	2.93	0.055
Can Read Food Labels	276	3.25 (±0.81)	21	3.29 (±1.06)	61	3.39 (±0.80)	194	3.21 (±0.78)	1.26	0.285
Can Set a Healthy Eating Goal	268	3.12 (±0.70)	21	3.48 (±0.93)	61	3.25 (±0.70)	186	3.03 (±0.65)	5.39	0.005

**Table 4:** Baseline Characteristics for SUYN Participants by Study Condition.

Variables	TOTAL	SUYN ONLY	Suyn + SO	*t* or χ^2^	*p*-Value
Age Group (*n* = 428)	74.71 (±11.45)	72.50 (±12.72)	77.98 (±8.29)	−5.39	<0.001
64 Years and Younger	58 (13.6%)	51 (19.9%)	7 (4.1%)	24.03	<0.001
65–69 Years	60 (14.0%)	37 (14.5%)	23 (13.4%)		
70–74 Years	70 (16.4%)	39 (15.2%)	31 (18.0%)		
75–79 Years	91 (21.3%)	52 (20.3%)	39 (22.7%)		
80 Years and Older	149 (34.8%)	77 (30.1%)	72 (41.9%)		
Malnutrition Risk Score (Screen II) (*n* = 429)	44.11 (±8.40)	44.65 (±8.55)	43.21 (±8.10)	1.75	0.081
No/Low	41 (9.6%)	30 (11.3%)	11 (6.7%)	8.73	0.013
Moderate	84 (19.6%)	61 (22.9%)	23 (14.1%)		
High	304 (70.9%)	175 (65.8%)	129 (79.1%)		
Fallen in Past 3 Months (*n* = 431)				1.46	0.228
No	342 (79.4%)	216 (81.2%)	126 (76.4%)		
Yes	89 (20.6%)	50 (18.8%)	39 (23.6%)		
Fear of Falling (*n* = 429)				13.65	0.003
Not At All	111 (25.9%)	81 (30.5%)	30 (18.4%)		
A Little	132 (30.8%)	82 (30.8%)	50 (30.7%)		
Somewhat	117 (27.3%)	58 (21.8%)	59 (36.2%)		
A Lot	69 (16.1%)	45 (16.9%)	24 (14.7%)		
Weight Changed in Past 30 Days (*n* = 324)				2.42	0.490
Yes, Gained	56 (17.3%)	35 (19.9%)	21 (14.2%)		
No, Stayed Same	211 (65.1%)	112 (63.6%)	99 (66.9%)		
Yes, Lost	49 (15.1%)	24 (13.6%)	25 (16.9%)		
Don’t Know	8 (2.5%)	5 (2.8%)	3 (2.0%)		
Eat Meals with Someone Daily (*n* = 329)				4.25	0.236
Almost Always	103 (31.3%)	63 (35.0%)	40 (26.8%)		
Often	45 (13.7%)	27 (15.0%)	18 (12.1%)		
Sometimes	126 (38.3%)	64 (35.6%)	62 (41.6%)		
Never/Rarely	55 (16.7%)	26 (14.4%)	29 (19.5%)		
Self-Described Appetite (*n* = 327)				3.02	0.221
Very Good	194 (59.3%)	106 (59.9%)	88 (58.7%)		
Fair/Good	120 (36.7%)	67 (37.9%)	53 (35.3%)		
Poor	13 (4.0%)	4 (2.3%)	9 (6.0%)		
Problems Getting Groceries (*n* = 321)				0.33	0.954
Never/Rarely	256 (79.8%)	141 (80.1%)	115 (79.3%)		
Sometimes	49 (15.3%)	26 (14.8%)	23 (15.9%)		
Often	8 (2.5%)	4 (2.3%)	4 (2.8%)		
Almost Always	8 (2.5%)	5 (2.8%)	3 (2.1%)		
Groceries Didn’t Last, Didn’t Have Money for More (*n* = 312)				2.09	0.351
Never	260 (83.3%)	137 (80.6%)	123 (86.6%)		
Sometimes	40 (12.8%)	25 (14.7%)	15 (10.6%)		
Often	12 (3.8%)	8 (4.7%)	4 (2.8%)		
Skipped Meals (*n* = 305)				3.36	0.186
Never	153 (50.2%)	92 (54.8%)	61 (44.5%)		
Sometimes	128 (42.0%)	63 (37.5%)	65 (47.4%)		
Often	24 (7.9%)	13 (7.7%)	11 (8.0%)		
Know Where to Get Resources, If Not Enough Money for Food (*n* = 275)				4.91	0.086
Often	84 (30.5%)	50 (32.3%)	34 (28.3%)		
Sometimes	52 (18.9%)	35 (22.6%)	17 (14.2%)		
Never	139 (50.5%)	70 (45.2%)	69 (57.5%)		
